# Reconstruction of atmospheric soot history in inland regions from lake sediments over the past 150 years

**DOI:** 10.1038/srep19151

**Published:** 2016-01-11

**Authors:** Y. M. Han, C. Wei, R.-J. Huang, B. A. M. Bandowe, S. S. H. Ho, J. J. Cao, Z. D. Jin, B. Q. Xu, S. P. Gao, X. X. Tie, Z. S. An, W. Wilcke

**Affiliations:** 1 KLACP and SKLLQG, Institute of Earth Environment, Chinese Academy of Sciences, Xi’an 710061, China; 2 Department of Environmental Science and Engineering, Xi’an Jiaotong University, Xi’an 710049, China; 3 Joint Center for Global Change Studies, Beijing 100875, China; 4 Laboratory of Atmospheric Chemistry, Paul Scherrer Institute (PSI), 5232 Villigen, Switzerland; 5 Institute of Geography, University of Bern, Hallerstrasse 12, 3012 Bern, Switzerland; 6 Oeschger Centre for Climate Change Research, University of Bern, Falkenplatz 16, 3012 Bern, Switzerland; 7 Desert Research Institute, 2215 Raggio Parkway, Reno, NV 89512, USA; 8 KLTECLSP, Institute of Tibetan Plateau Research, Chinese Academy of Sciences, Beijing 100101, China; 9 Institute of Geography and Geoecology, Karlsruhe Institute of Technology (KIT), Reinhard-Baumeister-Platz 1, 76131 Karlsruhe, Germany; 10 SCDRC, Shanghai Advanced Research Institute, Chinese Academy of Sciences, Shanghai 201210, China

## Abstract

Historical reconstruction of atmospheric black carbon (BC, in the form of char and soot) is still constrained for inland areas. Here we determined and compared the past 150-yr records of BC and polycyclic aromatic compounds (PACs) in sediments from two representative lakes, Huguangyan (HGY) and Chaohu (CH), in eastern China. HGY only receives atmospheric deposition while CH is influenced by riverine input. BC, char, and soot have similar vertical concentration profiles as PACs in both lakes. Abrupt increases in concentrations and mass accumulation rates (MARs) of soot have mainly occurred since ~1950, the establishment of the People’s Republic of China, when energy usage changed to more fossil fuel contributions reflected by the variations in the concentration ratios of char/soot and individual PACs. In HGY, soot MARs increased by ~7.7 times in the period 1980–2012 relative to the period 1850–1950. Similar increases (~6.7 times) were observed in CH. The increase in soot MARs is also in line with the emission inventory records in the literature and the fact that the submicrometer-sized soot particles can be dispersed regionally. The study provides an alternative method to reconstruct the atmospheric soot history in populated inland areas.

Black carbon (BC, sometimes also termed elemental carbon, EC), produced from incomplete combustion of fossil fuel and vegetation^1^,^2^,^3^,^4^, is a kind of specific carbonaceous aerosol that contributes substantially to the current bad air quality at many places in China. It occurs in different particle sizes that can enter the human lungs and contains many toxic compounds such as polycyclic aromatic compounds (PACs)^5^. Furthermore, BC, especially in the form of soot, absorbs sunlight and warms the Earth^6^,^7^. Even though BC is not the most abundant component in the atmosphere^8^,^9^, its climatic effects cannot be overlooked^4^,^6^,^10^. The long-term history of atmospheric BC is critical to understand its potential effects on human health and global climate.

Atmospheric science defines BC in different manner in comparison with soil and sediment science. In the atmospheric environment, BC is thought to be exchangeable with soot^4^, the part of submicron particle only formed in flame via gas-to-particle conversion^2^. Soil and sediment studies define BC as a combustion “continuum” ranging from char, the combustion residues produced by pyrolysis in smoldering fires, to refractory soot produced at higher temperatures in flames^2^. The definition of BC in atmospheric science^4^ is very different from its measurement^11^. Our recent test of standard reference materials demonstrated that both char and soot parts were included in the measurements of aerosol BC when measured with the most widely used thermal/optical method^12^. This finding is also consistent with other aerosol studies^13^,^14^. Thus, in this study we followed our previous definition^12^ of the two subtypes of BC, char and soot, and applied the thermal/optical method to differentiate them.

Presently, historical reconstruction of BC variations in the atmosphere are primarily available from remote areas such as high-latitude ice-covered regions^15^,^16^,^17^, where ice cores can be used to record atmospheric BC, especially the resulting soot deposition^15^. In addition, statistical data on fuel usage along with their combustion emission factors have been utilized to reconstruct the BC emissions regionally and globally^18^,^19^ to produce a rough indication of atmospheric BC history. However, the ice core reconstruction cannot reflect inland BC history, where BC emissions are dominated by sources related to urban and industrial activities. Moreover, the reconstructions of the long-term history from fuel usage are limited because of the unavailability of fuel usage data in the literature. Particularly, some important sources such as residential emissions from cooking and heating are difficult to estimate. Thus, more reliable measurements of atmospheric BC, especially soot emissions (and subsequent deposition to the land surface) and the reconstruction of its history are needed for inland areas^20^,^21^,^22^.

Lake sediments can record the long-term BC history from both atmospheric and river inputs in inland areas because BC is inert and resistant to degradation^1^; however, it is hard to differentiate atmospheric deposition from the river input. Efforts to reconstruct the atmospheric BC history via sediment records were conducted in remote areas^22^, but these results cannot be easily extended to more densely populated regions. Soot and char as the two subtypes of BC have different size fractions and thus different transport pathways^2^,^14^,^23^; the smaller soot fraction is atmospherically widely distributed, while the larger char fraction tends to be deposited close to the emission source. Consequently, soot can be far distributed in the atmosphere^2^ and thus more reliably reflects the combustion history at a regional scale than char. Thus, we hypothesize that lake sedimentary soot may be used to reflect its atmospheric deposition history. To test the hypothesis, the comparison of soot history from a common lake that receives riverine inputs with a “standard” archive that is almost exclusively driven by the deposition from the atmosphere, is needed. Huguangyan Maar lake (HGY) is suggested to uniquely receive atmospheric deposition with very limited inputs from other sources such as tributary rivers and soil erosion^24^ and thus can provide such “standard” archive for this study.

PACs are co-produced with BC from biomass and fossil fuel combustions^25^, and may even serve as precursors of soot formation in combustion processes^26^,^27^. Although some PACs such as polycyclic aromatic hydrocarbons (PAHs) have been extensively studied in lake sediments of China and worldwide^28^,^29^,^30^, very few studies have been extended to their derivatives such as oxygenated PAHs (OPAHs) and azaarenes (nitrogen heterocyclic PAHs, AZAs). Some of the derivatives have higher mutagenic and/or carcinogenic effects than their parent materials^31^. The polar PAC derivatives can be formed primarily by the combustion process itself or secondarily by reactions between parent-PAHs and oxidants such as ozone (O_3_), the hydroxyl radical (OH·) or NO_x_, photolysis and microbial degradation of parent-PAHs^31^. Consequently, the composition of the polar PAC mixture can change during long-range transport. Combined analysis of PACs, BC, char, and soot in lake sediments facilitates our identification of their origins, sources, transport, and fates, and can confirm the reliability of their reconstructed emission histories.

In this study, sedimentary BC, char, soot, and PACs histories covering the past ca. 150 years were reconstructed from two lakes of eastern China, HGY and Chaohu lake (CH). HGY has only a minor local tributary while CH is fed by several rivers in the inland. Both of the lakes are located in the most industrialized region of contemporary China. We used concentration ratios of char/soot and individual PACs to infer the changes of energy usage. Moreover, we hypothesized that the sedimentary records of char, soot, and PACs could reflect the start of the fast industrialization in China after the 1950s and the changes in type of energy sources from mainly wood burning in the pre-1950s to fossil fuels in the post-1950s. By comparing soot records of the two lakes, in addition to BC emission inventories in the literature, we demonstrated that the sedimentary record can be used to reconstruct atmospheric soot history in populated areas.

## Chronology dating

**Figure 1:**
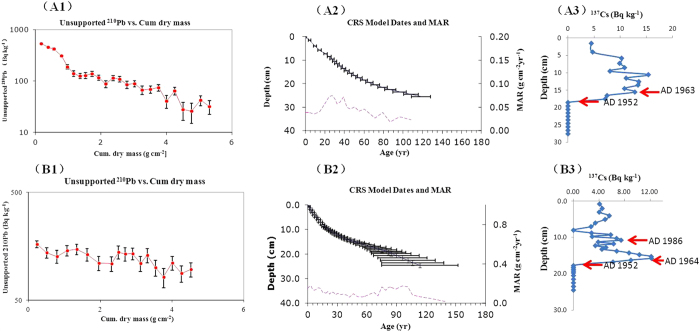
Chronology reconstruction from radionuclide activities of ^137^Cs, ^210^Pb and ^226^Ra for (**A**) the Huguangyan Maar Lake (HGY) and (**B**) the Chaohu Lake (CH). (A1 and B1) unsupported ^210^Pb versus cumulative dry mass; (A2 and B2) chronology reconstructed from the constant rate of supply (CRS) model (upper black line; horizontal lines indicating uncertainties) and the calculated mass accumulation rates (MARs, purple line); (A3 and B3) ^137^Cs profiles are in good agreement with the starting of global nuclear testing of ~1952-53 and the global nuclear fallout of ~1963-64 reconstructed by the CRS model.

The constant rate of supply (CRS) ^210^Pb model^32^,^33^ was applied for age dating of the two studied cores (Fig. 1). Also, ^137^Cs activities are presented as independent dates to confirm the CRS chronology (A3 and B3 in Fig. 1). The reconstructions with the CRS model for both cores precisely reflected the starting of global nuclear testing of ~1952–53 and the global nuclear fallout of ~1963–64, which are in good agreement with the ^137^Cs data. Furthermore, the small peak of the ^137^Cs activity at 10.75 cm in the CH core corresponds with the Chernobyl nuclear accident of 1986. We observed substantially lower mass accumulation rates (MARs) in HGY than in CH, which supports the dominant atmospheric deposition of particles in HGY. The ^137^Cs activities of the HGY core, however, did not decrease after the 1964 peak. A similar finding was reported for the Imitavika Lake in the Arctic^34^ and Sombre Lake in the Antarctic^35^ and attributed to a constant delivery of ^137^Cs to the lake sediments via atmospheric deposition and some suspended matters from glacier melt water. We therefore suggest that ^137^Cs is being continuously delivered to the HGY sediments by the atmospheric deposition and some soil erosion. As ^210^Pb has a half-life of 22.26 years, we can only produce reliable chronologies of 150–170 years. The dating of sediment layers older than 1850 were inferred from average accumulation rates of the upper layers. Consequently, our dating of the sediments older than 1850 is less reliable. Therefore, we refrained from discussing the older sediments in this paper.

### Historical variations in concentrations and MARs of BC, char, and soot

**Figure 2:**
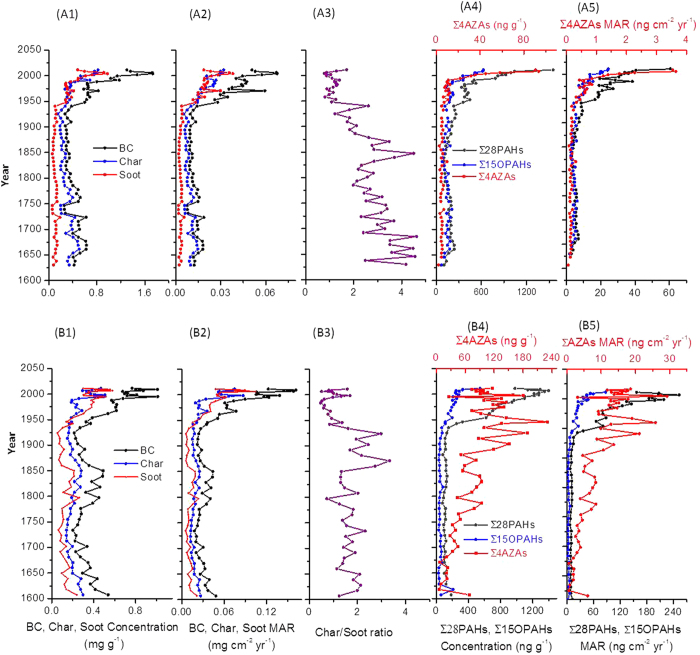
(Historical variations of concentrations and mass accumulation rates (MARs) of BC, char, and soot, char/soot ratios, parent-PAHs (∑28PAHs), oxygenated PAHs (∑15OPAHs), and azaarenes (∑4AZAs)in (**A**) the Huguangyan Maar Lake (HGY) and (**B**) the Chaohu Lake (CH). A1 and B1) BC, char, and soot concentrations; (A2 and B2) BC, char, and soot MARs; (A3 and B3) char/soot ratios; (A4 and B4) concentrations of parent-PAHs (∑28PAHs), oxygenated PAHs (∑15OPAHs), and azaarenes (∑4AZAs); (A5 and B5) MARs of ∑28PAHs, ∑15OPAHs, and ∑4AZAs. Note: The chronologies older than 1850 were obtained by assuming the average accumulation rate of the upper layers because the ^210^Pb method can only produce reliable chronologies of 150–170 years. Consequently, the older dates have a high uncertainty.

Historical variations of the concentrations and MARs of BC, char, and soot in HGY and CH showed an increasing trend from the bottom to the top of the two cores (Fig. 2). This is similar for all three target variables in both lake cores. Concentrations and MARs of BC, char, and soot sharply increased by the end of the 1940s and the beginning of the 1950s when the People’s Republic of China (PRC) was established after a long time of wars. Another obvious increase in BC, char, and soot concentrations and MARs happened in the late 1970s, when the economic reform and opening-up policy started in China. The highest concentrations and MARs of BC, char, and soot occurred in both lakes in the period 2004–2006, followed by a rapid decrease in recent years. This is in good agreement with the sulfur dioxide (SO_2_) emission peak in China, which also occurred in the period 2004–2006 and is considered as an indicator of coal combustion^36^. At that time the Atmospheric Pollution Prevention Act came into power and flue gas desulfurization in power plants was widely implemented^37^. At the same time the 11^th^ Five-Year Plan of China aimed at a reduction of SO_2_ (and associated BC) emissions per unit of energy consumption. The increases in BC, char, and soot concentrations in the late 1970s had already been reported in our previous study of sediments of the CH lake^38^; however, the strong increase in the 1950s had not been reported previously.

From pre-1850 to ~1930–40s, the overall trends of BC, char, and especially soot concentrations and MARs varied little in spite of a few peaks, which are comparable with those in 1950–1980 (Fig. 2). The lowest concentrations of BC and char occurred, although not notable, for the two cores in the same time interval (~1930s) when China fell into warfare.

### Similar vertical concentration trends of PACs with BC, char, and soot

The vertical trends of the concentrations of parent-PAHs (∑28PAHs, excluding perylene) and OPAHs (∑15OPAHs) paralleled those of BC (and their sub-fractions) in both lakes, indicating dominant human emissions for these pollutants and their co-emission from combustion sources. In a previous study, we found that PAHs concentrations are more closely correlated with those of soot in sediments than in soils, indicating that soot is a major sorbent of PAHs in sediments^39^. Our finding of strong correlations (r = 0.76–0.88, p < 0.001) of the concentrations of ∑28PAHs and ∑15OPAHs with those of soot in the HGY and CH sediment cores (Table S2) is in line with the previous observation. The correlations between PACs concentrations and those of BC were weaker in CH than in HGY, which may be attributable to a dilution effect from the river inputs into the lake.

However, there were also asynchronous developments of the concentrations of ∑28PAHs and BC fractions (Fig. 2). In the HGY core, the lowest ∑28PAHs occurred around the 1860s, the beginning of the Westernization Movement (WM), the pioneer industrialization period in China; however, the lowest soot concentrations were found around the 1930s. Moreover, in recent years (after the economic reforms starting in 1978), there were rapid increases in concentrations of ∑28PAHs, ∑15OPAHs, and ∑4AZAs (sum of the concentrations of four azaarenes) in HGY, whereas the concentrations of BC decreased after 2004–06 (Fig. 2). This indicates that the dominant factor determining the historical variations of PACs are their emission strengths, although their relationships with BC are enhanced by their sorption to co-emitted carbon fractions. Even though BC fractions and PACs are co-emitted from all combustion processes, each combustion source can generate different characteristic mixtures of carbonaceous matters and PACs^40^. PAHs are generally produced at relatively lower temperatures than soot and can also act as precursors of soot^26^. The dissimilar distribution patterns of ∑28PAHs, ∑15OPAHs, and ∑4AZAs in HGY and CH (Figs S2–S4) suggest that the two regions were influenced by different BC and PACs sources.

AZAs showed different vertical concentration distributions in the two lakes (Fig. 2). In HGY, the vertical distribution of ∑4AZAs paralleled those of the concentrations of BC fractions, ∑28PAHs and ∑15OPAHs, with two sharp increases at the beginning of the 1950s and in the late 1970s, respectively. However, in CH, ∑4AZAs concentrations increased from the pre-1850s to 1950 but thereafter decreased. We attribute the differences to the river inputs to CH and thus a specific local source enriched in AZAs. Our results demonstrate that HGY mainly received BC and PACs from the atmospheric input with some minor input from soil erosion immediately adjacent to the maar lake, while the input from the tributary rivers was a significant source of BC and PACs to the sediments of CH.

Previous studies have suggested that perylene in pristine sediments originates mainly from *in-situ* biogenic diagenesis under anoxic conditions^41^,^42^. In line with this, perylene in HGY had higher concentrations in the lower parts of the core (Fig. S5), which is opposite to other PACs. Relatively lower concentration and different vertical distribution pattern of perylene was found in CH compared to HGY (Fig. S5). We suggest that this is associated with the different oxygen concentrations in the two lakes. HGY with a water depth of ~20 m likely shows anaerobic conditions near the ground. For CH, the water depth is only ~2.7 m, and the water currents from the tributary and leaving rivers likely create aerobic conditions near the ground.

### Changing composition of fossil energy sources inferred from char/soot and PAC concentration ratios

Industrialization in the Western developed countries began in ~1750, the start of the Industrial Revolution. Although pioneer industrialization in China can be traced back to the 1860s (the beginning of the WM), accelerated industrialization occurred after the establishment of the PRC (1949–50). The char/soot ratio has been suggested to be indicative of the type of fuel combusted in urban areas. Biomass burning usually produces higher char/soot concentrations ratios while fossil fuel combustions, especially motor vehicle emissions, produce lower ratios^43^,^44^. There was a sharp decrease in char/soot ratios in both lakes in ~1950 (A3 and B3 in Fig. 1), supporting the increase in fossil fuel combustion contributions after the establishment of the PRC. However, in both lakes a small increasing trend of the char/soot ratio was observed since 1950. This is very likely due to the rapid increase in the population of eastern China in this time period (Fig. S6). The increasing population probably resulted in significantly increasing deposition of char into these lakes from multiple local sources related to the increased human activities. Abrupt increases in the MARs of retene further suggest increases in biomass burning (in both regions), which will also lead to increasing char/root ratios (Fig. S7).

**Figure 3:**
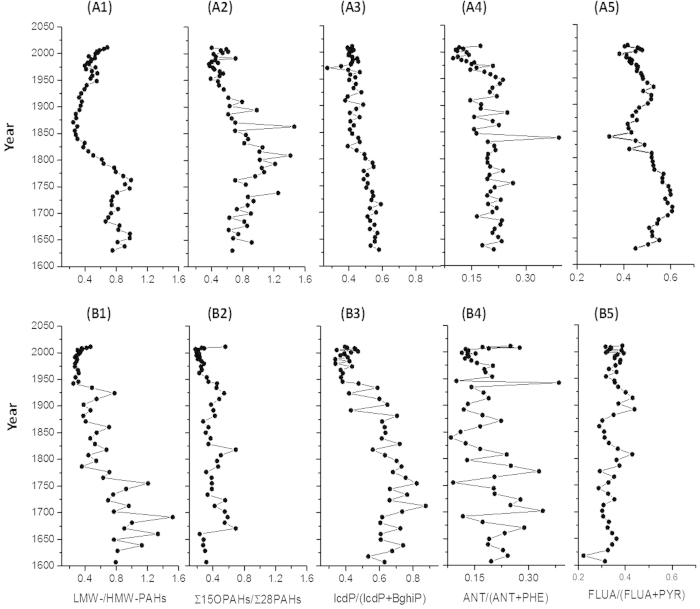
Vertical variations of the selected PAC concentration ratios for source identification in (**A**) the Huguangyan Maar Lake (HGY) and (**B**) the Chaohu Lake (CH). (A1 and B1) concentration ratio of low-molecular-weight (LMW) PAHs to high-molecular-weight (HMW) PAHs; (A2 and B2) ∑15OPAHs/∑28PAHs concentration ratios; (A3 and B3) concentration ratio of indeno[1,2,3-cd]pyrene (IcdP) to the sum of IcdP and benzo[g,h,i]perylene (BghiP); (A4 and B4) concentration ratio of anthracene (ANT) to the sum of ANT and phenanthrene (PHE); (A5 and B5) concentration ratio of fluoranthene (FLUA) to the sum of FLUA and pyrene (PYR). Note: Low- molecular-weight (LMW) PAHs include naphthalene, acenaphthylene, acenaphthene, fluorene, phenanthrene, and anthracene; High- molecular-weight (HMW) PAHs include fluoranthene, pyrene, benzo[a]anthracene, chrysene+triphenylene, benzo[b+j+k]fluoranthene, benzo[e]pyrene, benzo[a]pyrene, indeno[1,2,3-cd]pyrene, dibenzo[a,h]anthracene, benzo[g,h,i]perylene, and coronene. Note: The chronologies older than 1850 were obtained by assuming the average accumulation rate of the upper layers because the ^210^Pb method can only produce reliable chronologies of 150–170 years. Consequently, the older dates have a high uncertainty.

Trends of selected concentrations ratios of individual PACs may assist in tracking PACs sources^45^,^46^, although the composition of the PAH mixtures are potentially altered during transport in the atmosphere and after deposition to soils before being eroded into lakes. The concentration ratio of low-molecular-weight (LMW) to high-molecular-weight (HMW) PAHs (Fig. 3, see explanations in the footnote, similar as below) is the most common tool to reflect the relative contribution of biomass to fossil fuel^46^. This ratio had an overall decreasing trend from bottom to top of the CH core while the lowest value was found in the 1950s (Fig. 3). This is consistent with the char/soot ratio that proves the increase in the contribution of fossil fuel combustion in the 1950s. In HGY, the LMW-/HMW-PAHs concentration ratios were higher in the bottom than the top of the core; however, the lowest ratio was seen in the beginning of the WM (around 1860s) because HGY is located much closer to the southern countries. This finding suggests that the pioneer industrialization by the WM had released more toxic PAHs (HMW-PAHs) into the atmosphere than the later development, which is reflected by the low LMW-/HMW-PAHs concentration ratios.

Similar to the trends of LMW-/HMW-PAHs concentration ratios, ∑15OPAHs/∑28PAHs also decreased since ~1950 (Fig. 3). This further supports our conclusion that the combustion of fossil fuel which emits lower OPAHs-to-parent-PAH amounts (compared to combustion of biomass^47^,^48^) increased during and after the 1950s. Finally, the decreases in the concentration ratios of IcdP/(IcdP+BghiP), FLUA/(FLUA+PYR), and ANT/(ANT+PHE) (see explanations in footnote of Fig. 3) in the two lakes, more obviously in HGY, are in line with the significant increase in petroleum combustion^29^,^45^.

### Atmospheric vs. river debris inputs of pollutants

**Figure 4:**
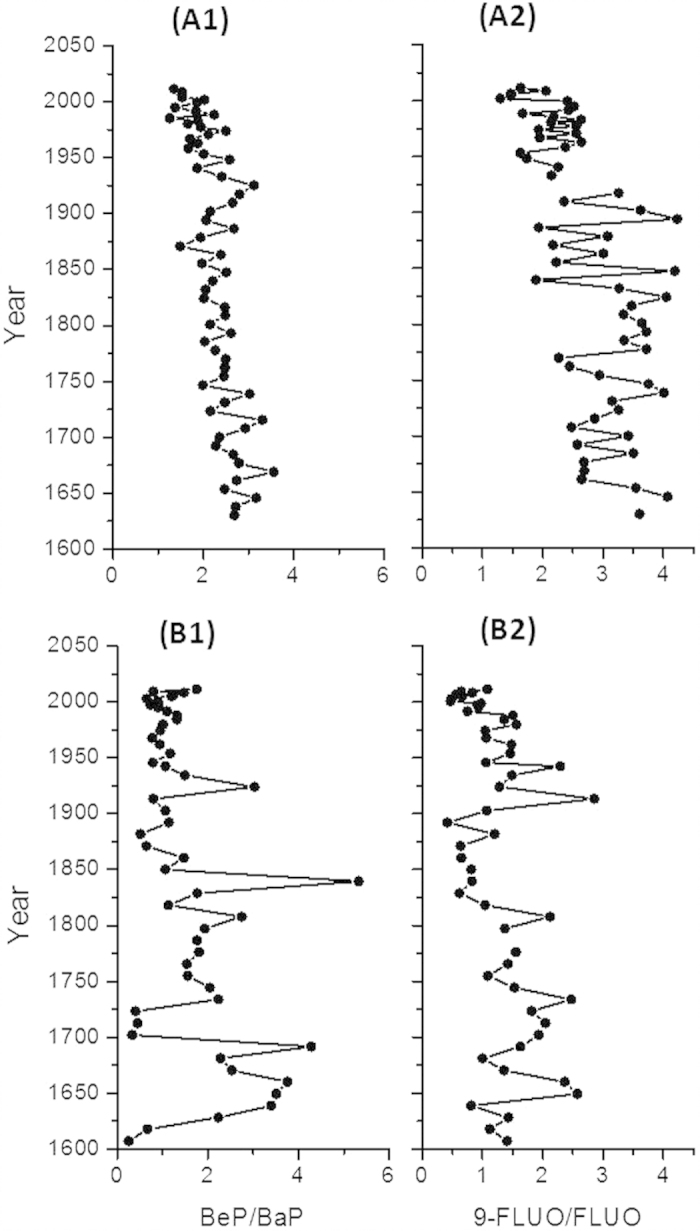
Concentration ratios of benzo(e)pyrene/benzo(a)pyrene (BeP/BaP) and 9-fluorenone/fluorene (9-FLUO/FLUO), which are used to indicate atmospheric transport and oxidation. Note: The chronologies older than 1850 were obtained by assuming the average accumulation rate of the upper layers because the ^210^Pb method can only produce reliable chronologies of 150–170 years. Consequently, the older dates have a high uncertainty.

The concentration ratios BeP/BaP and 9-FLUO/FLUO were higher in HGY than in CH sediments (Fig. 4). Because BaP is more susceptible to atmospheric degradation than BeP^47^,^49^ and 9-FLUO is one of the oxidation products of FLUO^50^,^51^, long-term exposure to atmospheric ultra-violet (UV) radiation would lead to higher BeP/BaP and 9-FLUO/FLUO concentration ratios. Therefore, we interpret the differences in BeP/BaP and 9-FLUO/FLUO concentrations ratios between the two lakes as an indication of the dominance of atmospheric deposition to HGY, while river input of PACs plays an important role in CH. In addition, as population and human activities in eastern China increased, it is reasonable that the contribution from local emissions in the HGY region has increased in recent years. This also explains the decreases in BeP/BaP and 9-FLUO/FLUO concentration ratios (Fig. 4).

In HGY, the highest MARs of soot, which occurred in recent years, is ~21.3 times of the lowest MARs of soot in the 1930s, and the MAR of soot in the period 1980–2012 is ~7.7 times that of the period 1850–1950 (Table 1). However, the average MAR of char in the period 1980–2012 is only ~3.6 times that of the period 1850–1950. These observed differences in the temporal trends of char and soot are an indication of a higher contribution of local sources to char than soot in HGY.

The different transport distances of char and soot can also explain their historical variations in CH. The average MAR of soot in the period 1980–2012 is 6.7 times that of the period 1850–1950 (Table 1), which represents only a slightly smaller ratio than in HGY. We attribute the lower MARs of soot to the dilution by soot-poor clastic material deposited into CH by the riverine inputs. It has been shown that the current soot concentrations in the atmosphere are similar regionally^14^,^23^ and thus presumably the current soot deposition to the lake sediments are similar at both locations as well. The consistent increases of MARs of soot in the two lakes between 1980–2012 and 1850–1950 suggest that soot MARs in sediments reflect the variations in atmospheric soot concentrations, which provides a new pathway to reconstruct atmospheric soot history for inland areas using lake sediments. However, in CH there was only a small increase of the char MARs in 1980–2012 compared with that in 1850–1950 (~1.8 times on average), supporting that local river inputs can dilute the concentrations of char. Husain *et al.*^22^ have also reconstructed the atmospheric BC history using a lake sediment record at Whiteface Mountain, New York. They determined BC concentrations with the thermal/optical NIOSH method, which has been suggested to measures BC fractions tending to more resemble refractory BC such as soot than the IMPROVE method used in our work^38^,^52^.

Because sedimentary soot originates mainly from regional atmospheric deposition, the ratio of ∑28PAHs concentrations to soot can be used to reflect the local-to-regional influences. Small variations in ∑28PAHs/soot in HGY confirm the mainly atmospheric deposition of PAHs and soot to the Maar lake, while the increasing trend of ∑28PAHs/soot ratio in CH after 1850 is likely associated with increasing contributions from the local human activities via riverine inputs (Fig. S8). The far higher ratios of ∑4AZAs and retene to soot in CH than in HGY further support this conclusion.

### Comparison of the soot deposition history with BC emission inventories in China

**Figure 5:**
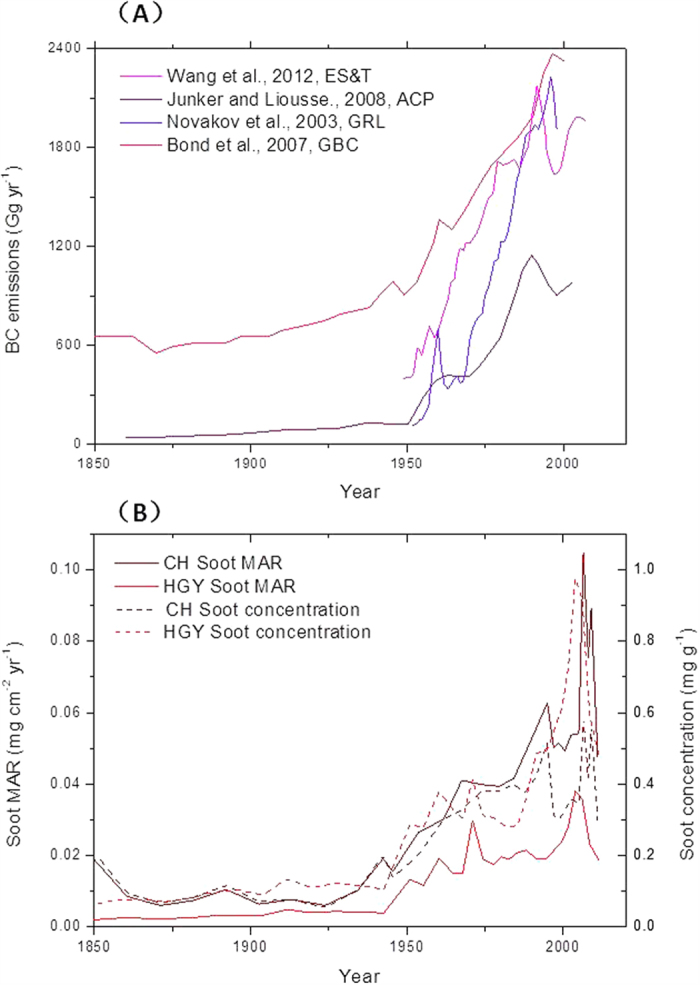
Comparison of (**A**) the black carbon emission inventory records in the literature^18^,^19^,^53^,^54^ and (**B**) our soot concentration and mass accumulation rate (MAR) records from the Huguangyan Maar Lake (HGY) and the Chaohu Lake (CH) showing comparable profiles.

The sedimentary record of soot concentrations in HGY and CH parallels the BC inventories for China^18^,^19^,^53^,^54^ (Fig. 5). The slight deviations between our lake soot records and the emission inventories of different groups^18^,^19^,^53^,^54^ underline that there may be local modifications of the national deposition patterns. Thus, we suggest that lake sediments are a reliable archive of the atmospheric soot history.

The available studies using ice cores to reconstruct the soot deposition history in China demonstrated a ~2.5 times increase in soot deposition between the pre-1950s and the post-1980s^55^,^56^, which is far lower than that of our sediment data (~6.7–7.7 times). This suggests that the long-range transport of soot in remote areas may decrease the influence of human activities, and thus ice core records cannot document the atmospheric soot history in populated areas.

The concentrations of PAHs, OPAHs, and AZAs showed similar vertical distributions as the MARs of soot (Fig. S9). However, the rates of increase in MARs of PACs and soot in the two regions were different (Table 1). It is likely that PACs in the two lakes originated mainly from local/regional emission sources, which explains the differences between the two lakes (Figs S2–S4). The higher increase rates of PACs concentrations in CH than in HGY sediments may indicate the significant additional input of PACs into CH by the riverine inputs. In contrast, the HGY catchment receives PACs only from atmospheric input. The atmospheric deposition of PAHs in HGY parallels their emission inventories^57^,^58^ (Fig. S9), further supporting our suggestion that the Maar lake provides a robust PACs history. Because there is a lack of long-term OPAHs and AZAs inventories in China, our results provide the first historical record of atmospheric OPAHs and AZAs concentration variations at inland regions of China.

### Implications of the reconstruction of the history of atmospheric pollutants

The differentiation between char and soot provides a new way to reconstruct the atmospheric soot history. This may be suitable for much longer-term atmospheric soot reconstruction such as for the past 2000 years, the Holocene, and the glacial-interglacial cycles. The atmospheric soot reconstruction in this study provides a robust record, which can be used to test BC emission inventories obtained from fuel usages. An abrupt increase in soot concentrations occurred in ~1950 in China, when more fossil fuels were used (Fig. S6). The timing is far later than that in the western developed countries, where the peak in atmospheric BC was found in the 1910s^16^ while more recently the BC deposition decreased in Europe since the 1960s^59^. The change of energy sources and the introduction of emission control techniques resulted in changes in concentration and composition patterns of the atmospheric BC and PACs. The notable decrease in soot MARs after 2004–06, which deviates from previous emission inventories obtained from fuel usages^37^, provides evidences of the important effects of environmental protection measures implemented by the Government of China.

Although both char and soot are light-absorbing, they have different optical properties. Char absorbs visible light weakly and tends to absorb short-wavelength light more strongly, while the light absorption capacity of soot is strong^44^. The increase in char and especially more soot emissions to the atmosphere associated with the change to fossil fuel usage may result in greater climatic warming effects. However, the climate system is complicated. For example, the composition of carbonaceous aerosol as characterized by the contributions of organic carbon (OC) and BC may vary among different energy source emissions. Because the OC/BC ratios have been suggested to indicate the warming or cooling effects of carbonaceous aerosols^60^, the change from biomass burning to fossil fuel combustion such as that occurring in 1950 seems likely to lead to more warming effects. However, more aerosol emissions from fossil fuel consumption, especially those of light-scattering particles such as sulfate would result in cooling effects. Therefore, understanding the effects of the various compounds emitted to the atmosphere by fossil fuel combustion requires knowledge of the histories of different atmospheric chemical species including trace gases and aerosols and their direct and indirect climatic radiative forcing. Our suggested new method for the reconstruction of the atmospheric soot history for inland regions contributes to closing the knowledge gap.

## Method

We selected Huguangyan Maar lake (HGY) and Chaohu lake (CH) in the developed regions of eastern and southeastern China, respectively, for this study (Fig. S1). HGY is located in Guangdong province and has a water area of ~2.5 km^2^ with an average water depth of ~20 m, and there are no river inputs and outlets. CH is located in Anhui Province and has a water area of ~775 km^2^ with a water depth of ~2.7 m, and there are eight larger tributary rivers and one outlet to Yuxi River, which is a tributary of the Yangtze River. Two sediment cores were obtained using a gravity corer from HGY (HGY12-2) and CH (CH12-3), respectively, in January 2012. The cores were sectioned into 0.5-cm intervals of the upper 20 cm and 1.0-cm intervals of the lower part.

All samples were freeze-dried. Radionuclide concentrations of ^137^Cs, ^210^Pb and ^226^Ra were analyzed for dating by direct gamma counting of 3–6 g of dried sediments using a Canberra HpGe well detector model GCW2022. BC, char, and soot concentrations were quantified using the thermal/optical method with the IMPROVE protocol after acid pretreatment^12^,^61^. Furthermore, the concentrations of 29 parent- and alkyl-PAHs (including perylene), 15 OPAHs, and 4 AZAs^47^,^62^,^63^ were measured in the samples. Details of the locations, sampling, dating, analytical procedures and quality assurance/control are explained in the Supplementary Online Materials (SI). A summary of the concentrations of carbon fractions and PACs as well as their abbreviations are presented in Table S1.

## Additional Information

**How to cite this article**: Han, Y. M. *et al.* Reconstruction of atmospheric soot history in inland regions from lake sediments over the past 150 years. *Sci. Rep.* **6**, 19151; doi: 10.1038/srep19151 (2016).

## Supplementary Material

Supplementary Information

## Figures and Tables

**Table 1:** Summary of concentrations and mass accumulation rates (MARs) of black carbon (BC), char, and soot, as well as of parent-PAHs (∑28PAHs), oxygenated PAHs (∑15OPAHs), and azaarenes (∑4AZAs) in the Huguangyan Maar Lake (HGY) and the Chaohu Lake (CH) in different periods.

	Concentration (mg g^−1^)			MARs (μg cm^−2^ yr^−1^)			Concentration (ng g^−1^)			MAR (ng cm^−2^ yr^−1^)		
	BC	Char	Soot	BC	Char	Soot	∑28PAHs	∑15OPAHs	∑4AZAs	∑28PAHs	∑15OPAHs	∑4AZAs
Lake Huguangyan Maar (HGY)												
1980–2012	1.18 ± 0.38	0.62 ± 0.18	0.56 ± 0.23	48 ± 9	25 ± 4	23 ± 7	806.9 ± 335.6	406.6 ± 161.6	38.3 ± 30.9	35.0 ± 10.5	17.4 ± 4.5	1.6 ± 1.1
1950–1980	0.66 ± 0.07	0.34 ± 0.04	0.32 ± 0.05	34 ± 10	17 ± 5	17 ± 5	387.9 ± 33.5	174.7 ± 31.2	9.4 ± 2.1	21.1 ± 3.7	9.4 ± 1.6	0.5 ± 0.1
1850–1950	0.31 ± 0.03	0.21 ± 0.03	0.10 ± 0.02	10 ± 2	7 ± 1	3 ± 1	194.7 ± 46.4	134.6 ± 41.9	5.1 ± 4.9	6.7 ± 2.0	4.6 ± 1.7	0.17 ± 0.1
pre–1850	0.43 ± 0.11	0.32 ± 0.09	0.11 ± 0.03	13 ± 3	9 ± 3	3 ± 1	158.0 ± 42.8	138.4 ± 50.3	5.0 ± 2.2	4.7 ± 1.2	4.2 ± 1.4	0.15 ± 0.1
Highest/Lowest	6.7	5.5	16.4	8.7	6.0	21.3	16.1	9.1	44.0	20.3	12.3	59.2
(1980-2012)/(1850-1950)	3.8	2.9	6.0	4.8	3.6	7.7	4.2	3.1	8.0	5.2	3.8	9.3
Lake Chaohu (CH)												
1980–2012	0.76 ± 0.14	0.36 ± 0.11	0.40 ± 0.10	116 ± 30	56 ± 20	60 ± 19	1174.5 ± 148.8	283.7 ± 88.2	108.5 ± 40.1	179.3 ± 44.0	43.5 ± 17.0	14.4 ± 6.6
1950–1980	0.55 ± 0.10	0.23 ± 0.04	0.32 ± 0.07	62 ± 10	26 ± 5	35 ± 7	706.9 ± 69.1	187.9 ± 26.5	114.0 ± 30.8	79.3 ± 8.0	21.3 ± 4.2	12.9 ± 4.1
1850–1950	0.30 ± 0.08	0.20 ± 0.05	0.10 ± 0.05	28 ± 7	19 ± 4	9 ± 4	118.3 ± 18.7	47.5 ± 14.0	99.4 ± 46.7	11.3 ± 2.4	4.6 ± 1.8	9.6 ± 5.3
pre–1850	0.34 ± 0.09	0.20 ± 0.05	0.14 ± 0.05	31 ± 8	18 ± 5	12 ± 5	113.0 ± 22.8	54.1 ± 36.8	45.8 ± 28.4	9.7 ± 2.9	4.9 ± 3.3	4.1 ± 2.6
Highest/Lowest	5.0	3.6	10.8	8.9	6.9	19.3	17.9	19.4	36.9	35.9	34.5	106.6
(1980-2012)/(1850-1950)	2.5	1.8	4.0	4.1	2.9	6.7	9.9	6.0	2.4	15.9	9.5	1.5
