# Supplementary material for: Reconstruction of atmospheric soot history in inland regions from lake sediments over the past 150 years
Source: Sci Rep. 2016 Jan 11;6:19151. doi: 10.1038/srep19151 (PMC4707497; doi:10.1038/srep19151)
Supplement: Supplementary Information [file srep19151-s1.pdf]

Supporting Online Materials for

Reconstruction of atmospheric soot history in inland regions from lake sediments over the past 150 years

Y.M. Han*, C. Wei, R.J. Huang, B.A.M. Bandowe, S.S.H. Ho, J.J. Cao, Z.D. Jin, B.Q. Xu, S.P. Gao, X.X. Tie, Z.S. An, W. Wilcke

* To whom correspondences should be addressed. E-mail address: yongming@ieecas.cn

This file is 21 pages in total, including materials listed below:

Materials and methods;

Figures S1 to S10;

Tables S1 to S2.

Materials and methods

Study area

Chaohu Lake (CH; 117º16′54″–117º51′46″E, 31º25′28″–31º43′28″ N) is one of the famous "five freshwater lakes" in Anhui Province of the eastern China, which has a catchment of ~12,900 km^2 and water area of 834 km^2. It's average water depth is ~2.7 m. Annual average air temperature is ~15-16 ℃, and annual average precipitation is ~ 1,100 mm yr^{-1}. Approximately 33 rivers supply water to the lake [1]. Quaternary clay dominates the recent sediments in the drainage basin, and there are outcrops of Devonian red quartz sandstone and Cretaceous white sandstone along the shore.

Huguangyan Maar Lake (HGY; 110º17′ E, 21º9′ N) is the largest wet Maar lake in the Zhanjiang city, Guangdong Province of the south eastern China, which has a catchment of ~3.5 km^2 and water area of ~2.5 km^2. It's average water depth is ~20 m and the largest depth can reach to ~40 m. Annual average air temperature is ~23 ℃, and annual average precipitation is ~ 1,600 mm yr^{-1}. Both of the two lakes are influenced by the Eastern Asian monsoon and thus the precipitation concentrates in summer. There is no inlet and the outlet to the lake and thus it only receives atmospheric deposition.

Sample collection and chronology reconstruction

In February 2012, a 102-cm long sediment core HGY12-2 was collected from the southwestern part of the HGY with a water depth of ~16 m using a gravity corer (Uwitec, Austria; Fig. S1C). A distinct water-sediment interface and discernible

laminations were observed, indicating a lack of bioturbation and post-depositional disturbance. The core was sectioned continuously at 0.5 cm intervals for the upper 20 cm, and then transferred to 1 cm interval.

Also, in February 2012, a 83-cm long sediment core CH12-3 was collected from the western part of the CH with a water depth of ~2.7 m using a gravity corer (Uwitec, Austria; Fig. S1B). The core was also sectioned continuously at 0.5 cm intervals for the upper 20 cm, and then transferred to 1 cm interval.

A distinct water-sediment interface and discernible laminations were observed in both cores, indicating a lack of bioturbation. All samples were freeze-dried, agate mortar ground, and frozen at -20 ℃ until further analysis. The water content and dry density data were estimated from the mass of samples before and after freeze-drying.

The activities of ^{137}Cs, ^{210}Pb, and ^{226}Ra were analyzed for sediment dating by direct gamma counting of 3-6 g of dried sediments using a multi-channel γ-ray spectrometer (PerkinElmer, GWL-120-15) [2,3] for both cores. The constant rate of ^{210}Pb supply (CRS) model[4-6], which is not influenced by the sediment fluxes, was applied for chronology reconstruction, and the reconstructed dates and their corresponding mass accumulation rates (MARs) were presented in Fig. 1. ^{137}Cs activities as the independent marker were compared with the reconstructed chronology and the both methods showed very close chronology reconstruction.

BC, char, and soot measurement

The IMPROVE (Interagency Monitoring of Protected Visual Environments) method was used to quantify BC (also termed elemental carbon, EC), char, and soot

concentrations following Han et al. [7,8]. Briefly, ~50 ±10 mg sediment for each sample was stepwise pretreated with hydrochloric acid, a mixture of hydrochloric and hydrofluoric acids, and hydrochloric acid to remove carbonate, metal oxides, and silicates, and then filtered through pre-baked (850 ℃ for 3 hours) quartz-fiber filters (0.4 μm pore size, Whatman) using deionized water and air dried in an oven (35 ℃ for 8 hours). A DRI Thermal/Optical Carbon Analyzer (Model 2001, Atmoslytic Inc. Calabasas, CA, USA) was used to implement the IMPROVE protocol. It reports four OC fractions (OC1 to OC4 at 120, 250, 450 and 550 ℃ in a pure helium atmosphere), three EC fractions (EC1 to EC3 at 550, 700 and 800 ℃ in 2% oxygen/98% helium atmosphere), and one pyrolyzed organic carbon (POC) fraction, which is monitored by a laser. The IMPROVE protocol defined BC (or EC) as the sum of the three EC fractions minus POC[9]. Han et al. [10] defined char as EC1 minus POC, while soot as the sum of EC2 and EC3.

The carbon analyzer was calibrated daily with known quantities of methane. Replicate analyses were performed at the rate of one per group of 10 samples. The difference in comparison with the average values from replicate analyses was < < 10% for BC, char and soot.

PAHs, OPAHs and AZAs analyses

PACs, including 29 parent- and alkyl-PAHs, 15 oxygenated-PAHs (OPAHs) and 4 azaarenes (AZAs) were extracted by accelerated solvent extractor (ASE 200: Dionex, Sunnyvale, CA, USA), followed by clean-up/fractionation using column chromatography (10 % deactivated silica gel), and detection and quantification of

compounds by 7890A gas chromatograph coupled to a 5975C mass spectrometer (GC/MS, Agilent, Santa Clara, CA, U.S.A.) [11,12].

Firstly, about 2 g of sediment sample were weighed and placed in the 33 mL ASE extraction cells, spiked with 7 deuterated-PAHs (naphthalene-D_8, acenaphthene-D_{10}, phenanthrene-D_{10}, pyrene-D_{10}, chrysene-D_{12}, perylene-D_{12}, and benzo[g,h,i]perylene-D_{12}) and 2 deuterated-OPAHs (benzophenone-D_5 and anthraquinone-D_8) as internal standards. Extra space in the ASE cell was filled with an inert sorbent (Isolute HM-N, Biotage, Upsala, Sweden). Target compounds were then extracted by pressurized liquid extraction using an ASE 200. During extraction, the cells were filled with dichloromethane (DCM), pressurized to 14 MPa, and heated to 120 ℃ within 6 min. Temperature and pressure of the ASE cells were then held constant for 5 min, and finally eluted with more DCM purging with N_2 for 90 s. The extraction cycle for each sample was repeated a second time. The two extracts from each sample were combined, dried with sodium sulfate, rotary evaporated (35 ℃), and solvent exchanged to hexane.

Secondly, 8 mL borosilicate glass columns (J.T. Baker, Center Valley, PA, USA) were packed with 3 g of silica gel (10% deactivated). Packed columns were eluted by 10 mL hexane (HEX) before loading samples. The *a* fraction, containing alkyl- and parent PAHs was eluted by 15 mL hexane: dichloromethane (HEX: DCM, 5:1, v/v), the *b* fraction containing AZAs and OPAHs was eluted by 8 mL DCM followed by 5 mL acetone. Five drops of toluene were added to *a* and *b* fractions, respectively and the solvent was evaporated to about 0.5 mL. 25 uL fluoranthene-D_{10} (20 ng μL^{-1} as

recovery standard) was then added to the final solutions before being transferred to 2 mL GC-vials for GC/MS measurements.

Thirdly, separation and quantification of PACs was performed with an Agilent 7890A GC system equipped with a 7963A auto-sampler, split/splitless injector and inert mass spectrometer 5975C with triple axis detector. The GC was equipped with a DP-5MS column (5% phenyl-95% methyl-polysiloxane, 30 m long × 0.25 mm diameter × 0.25 μm film thickness). Helium was used as carrier gas with a constant flow rate of 1.2 mL min^{-1}. For measurement of the PAHs, AZAs and OPAHs, the instrumental parameters were set as follows: 1 μL injection volume (splitless mode), injection port temperature: 280 ℃, transfer line temperature: 310 ℃, ion source temperature: 230 ℃, and quadrupole temperature: 150 ℃. The oven temperature program for measuring PAHs started at 80 ℃ for 4 min. The temperature was increased to 160 ℃ at a rate of 14 ℃ min^{-1}, held for 1.5 min, increased to 225 ℃ at a rate of 5 ℃ min^{-1}, held for 5 min, and increased to a final temperature of 300 ℃ at a rate of 5 ℃ min^{-1} and held for 11 min [12]. For OPAH analysis, the initial oven temperature started at 80 ℃, held for 6 min, increased to 145 ℃ at a rate of 5 ℃ min^{-1}, held for 3 min, increased to 200 ℃ at a rate of 5 ℃ min^{-1} and held for 7 min, increased to 300 ℃ at a rate of 15 ℃ min^{-1} and held for 5 min [12]. Target compounds were quantified by the internal standard technique using seven calibration standards prepared from target compound standards each spiked with a constant concentration of internal standard.

All glassware used in this study was rinsed with acetone, machine-washed,

baked at 250 ℃ for 12 h, and rinsed with high purity solvents before use. All solvents used for extraction, column chromatography, and standard preparation were high purity for pesticide residue analysis. To check and correct for possible contaminations during the analytical procedure, we processed blanks made of diatomaceous earth at the beginning of each batch of 24 samples. Most compounds were either not detected in the blanks or were measured in negligible quantities, significantly below those found in the samples. The measured compound concentrations in the samples were corrected by subtracting the mean blank concentrations. We checked the accuracy of PAH measurements by including the certified reference material ERM-CC013a (BAM, Berlin, Germany) into our analyses. The mean recovery of all PAHs for which certified values were provided was 97% (58%-130%). We checked the recovery of all internal standards. The mean recovery of deuterated PAHs and OPAHs was 88% (63-113%) and 108% (95%-120%), respectively.

Mass accumulation rate (MAR) calculation

As the ^{210}Pb CRS reconstructed chronology reported the mass accumulation rate (MAR) for each measured sample, it is directly used for the calculation of MARs for any pollutant used in this study. The MARs were calculated using the formula listed below:

Pollutant MAR = pollutant concentration * bulk MARs (1)

References

1 Wang, S. Y., Jin, C. S., Meng, R. X. & Xu, F. L. *Chaohu Lake in Anhui Province, China*. (Haiyang Press, 1995).

2 Jin, Z., Han, Y. & Chen, L. Past atmospheric Pb deposition in Lake Qinghai, northeastern Tibetan Plateau. *J. Paleolimnol.* **43**, 551-563, doi:10.1007/s10933-009-9351-6 (2010).

3 Han, Y. M. *et al.* Distribution and ecotoxicological significance of trace element contamination in a similar to 150 yr record of sediments in Lake Chaohu, Eastern China. *J. Environ. Monitor.* **13**, 743-752, doi:10.1039/c0em00551g (2011).

4 Appleby, P. in *Tracking environmental change using lake sediments* (eds W.M. Last & J.P. Smol) 171-203 (Springer, 2001).

5 Appleby, P. G. Three decades of dating recent sediments by fallout radionuclides: a review. *Holocene* **18**, 83-93, doi:10.1177/0959683607085598 (2008).

6 von Gunten, L. *et al.* Age modeling of young non-varved lake sediments: methods and limits. Examples from two lakes in Central Chile. *J. Paleolimnol.* **42**, 401-412, doi:10.1007/s10933-008-9284-5 (2009).

7 Han, Y. N. *et al.* Evaluation of the thermal/optical reflectance method for quantification of elemental carbon in sediments. *Chemosphere* **69**, 526-533, doi:10.1016/j.chemosphere.2007.03.035 (2007).

8 Han, Y. M. *et al.* The effect of acidification on the determination of elemental carbon, char-, and soot-elemental carbon in soils and sediments. *Chemosphere* **75**, 92-99, doi:10.1016/j.chemosphere.2008.11.044 (2009).

9 Chow, J. C. *et al.* The dri thermal/optical reflectance carbon analysis system: description, evaluation and applications in U.S. Air quality studies. *Atmospheric Environment. Part A. General Topics* **27**, 1185-1201 (1993).

10 Han, Y. M. *et al.* Evaluation of the thermal/optical reflectance method for discrimination between char- and soot-EC. *Chemosphere* **69**, 569-574, doi:10.1016/j.chemosphere.2007.03.024 (2007).

11 Bandowe, B. A. M., Shukurov, N., Kersten, M. & Wilcke, W. Polycyclic aromatic hydrocarbons (PAHs) and their oxygen-containing derivatives (OPAHs) in soils from the Angren industrial area, Uzbekistan. *Environ. Pollut.* **158**, 2888-2899, doi:10.1016/j.envpol.2010.06.012 (2010).

12 Bandowe, B. A. M. & Wilcke, W. Analysis of Polycyclic Aromatic Hydrocarbons and Their Oxygen-Containing Derivatives and Metabolites in Soils. *Journal of Environmental Quality* **39**, 1349-1358, doi:10.2134/jeq2009.0298 (2010).

13 Zhang, Y. *et al.* in *Environmental Challenges in the Pacific Basin* Vol. 1140 *Annals of the New York Academy of Sciences* (ed D. O. Carpenter) 218-227 (2008).

14 Shen, H. *et al.* Global time trends in PAH emissions from motor vehicles. *Atmos. Environ.* **45**, 2067-2073, doi:10.1016/j.atmosenv.2011.01.054 (2011).

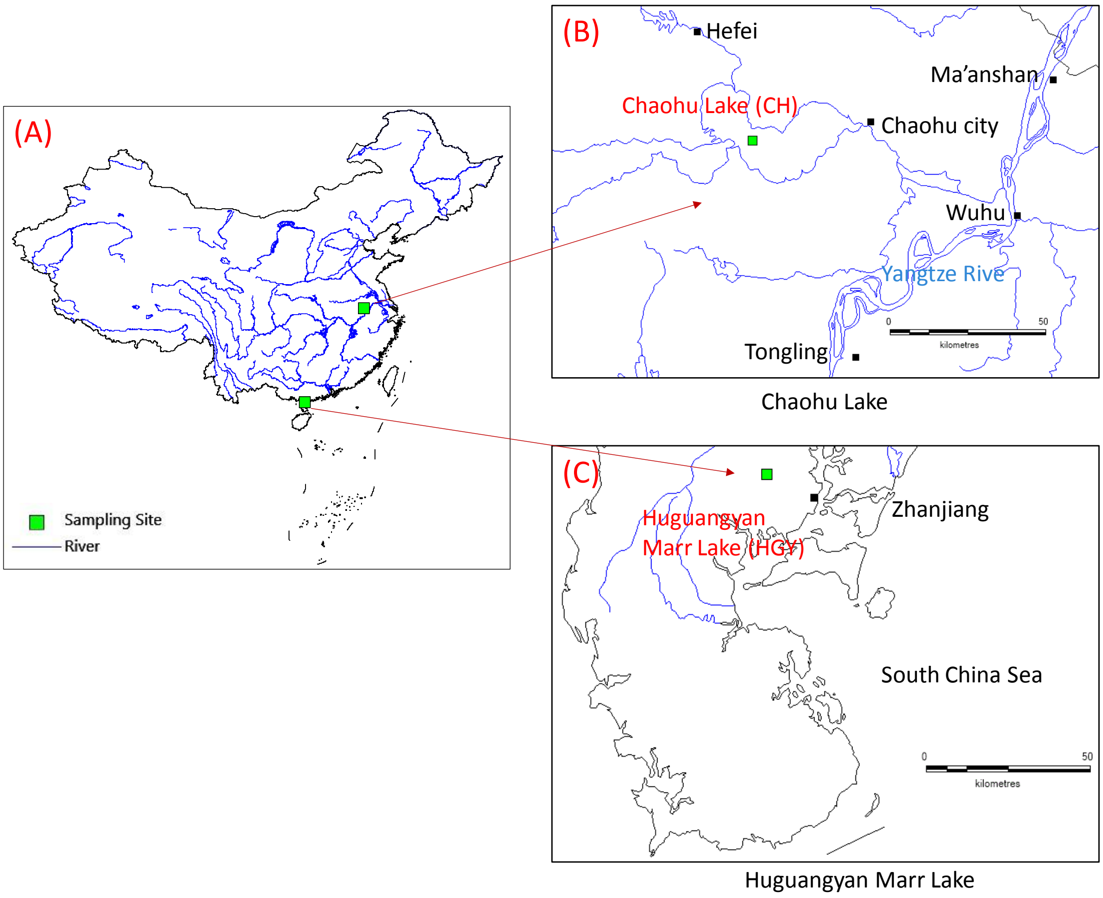

Fig. S1 Sampling locations of the Huguangyan Maar Lake (HGY) and the Chaohu Lake (CH). All the three figures were created with the software of QGIS version 2.12.0 (Open Source Geospatial Foundation Project, http://qgis.osgeo.org). These data for the figures were downloaded from Natural Earth (www.naturalearthdata.com).

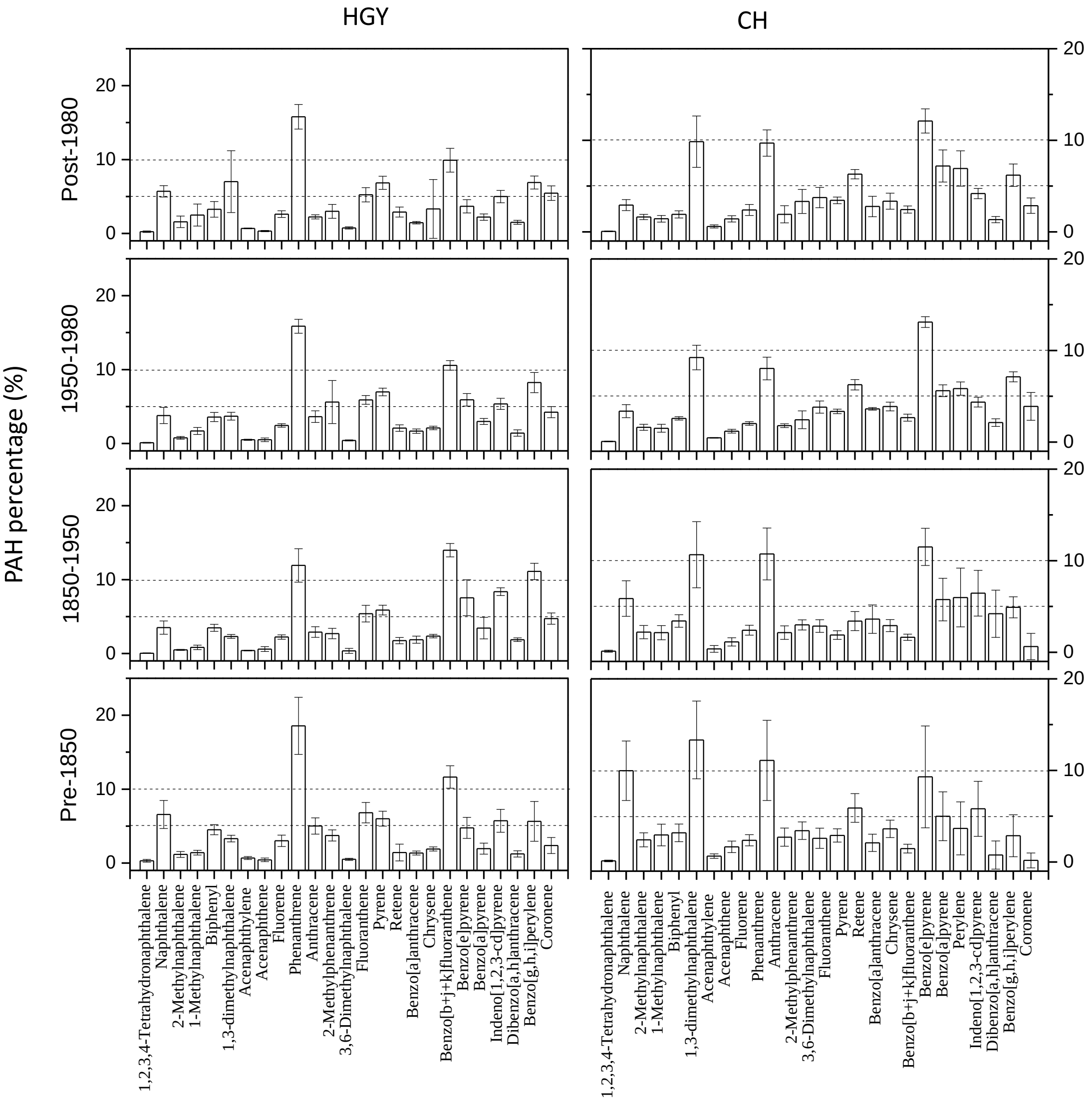

Fig. S2 Parent-PAHs distribution patterns in different periods for Huangyan Maar Lake (HGY) and Chaohu Lake (CH), respectively.

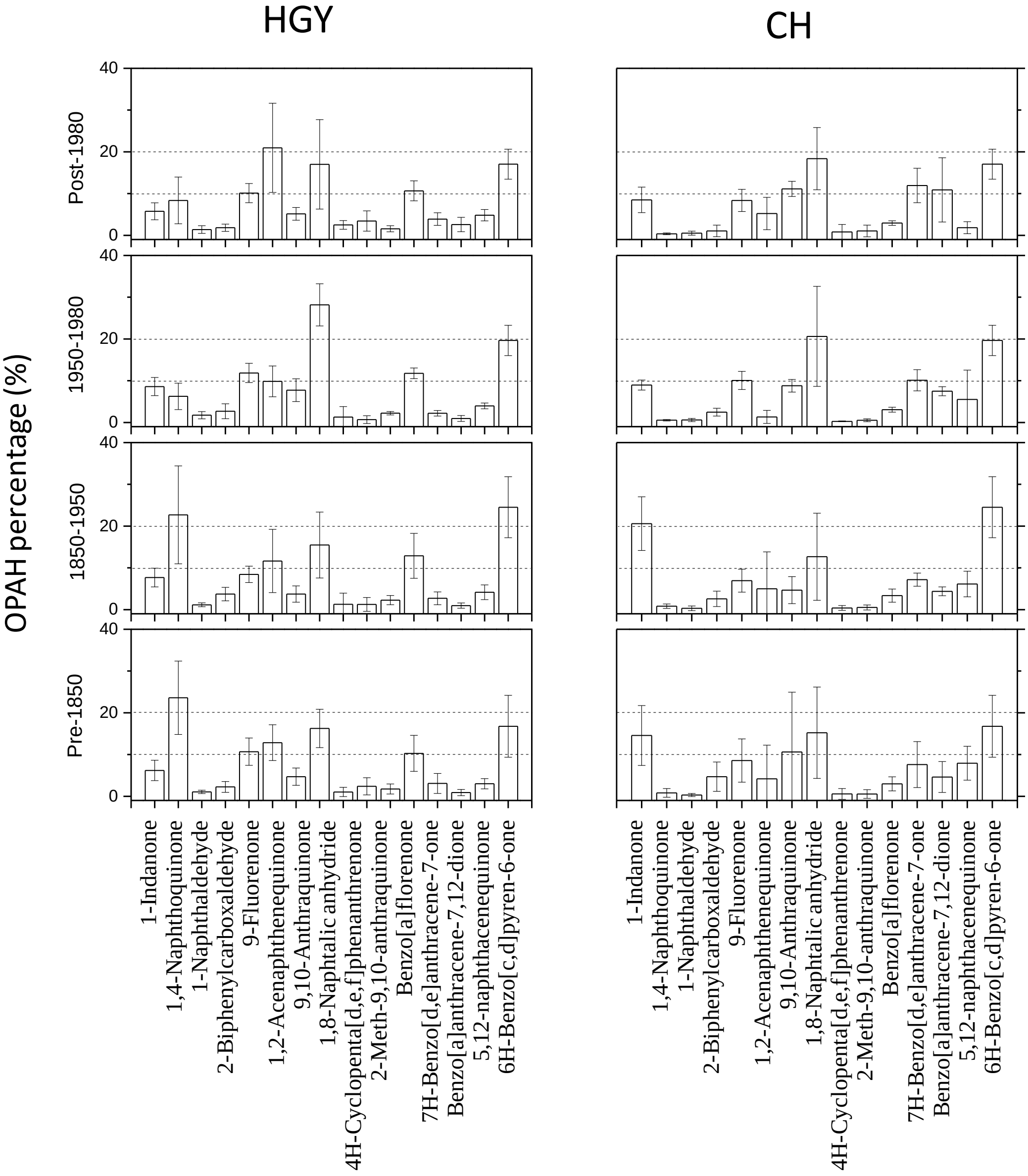

Fig. S3 Oxygenated-PAHs distribution patterns in different periods for Huangyan Maar Lake (HGY) and Chaohu Lake (CH), respectively.

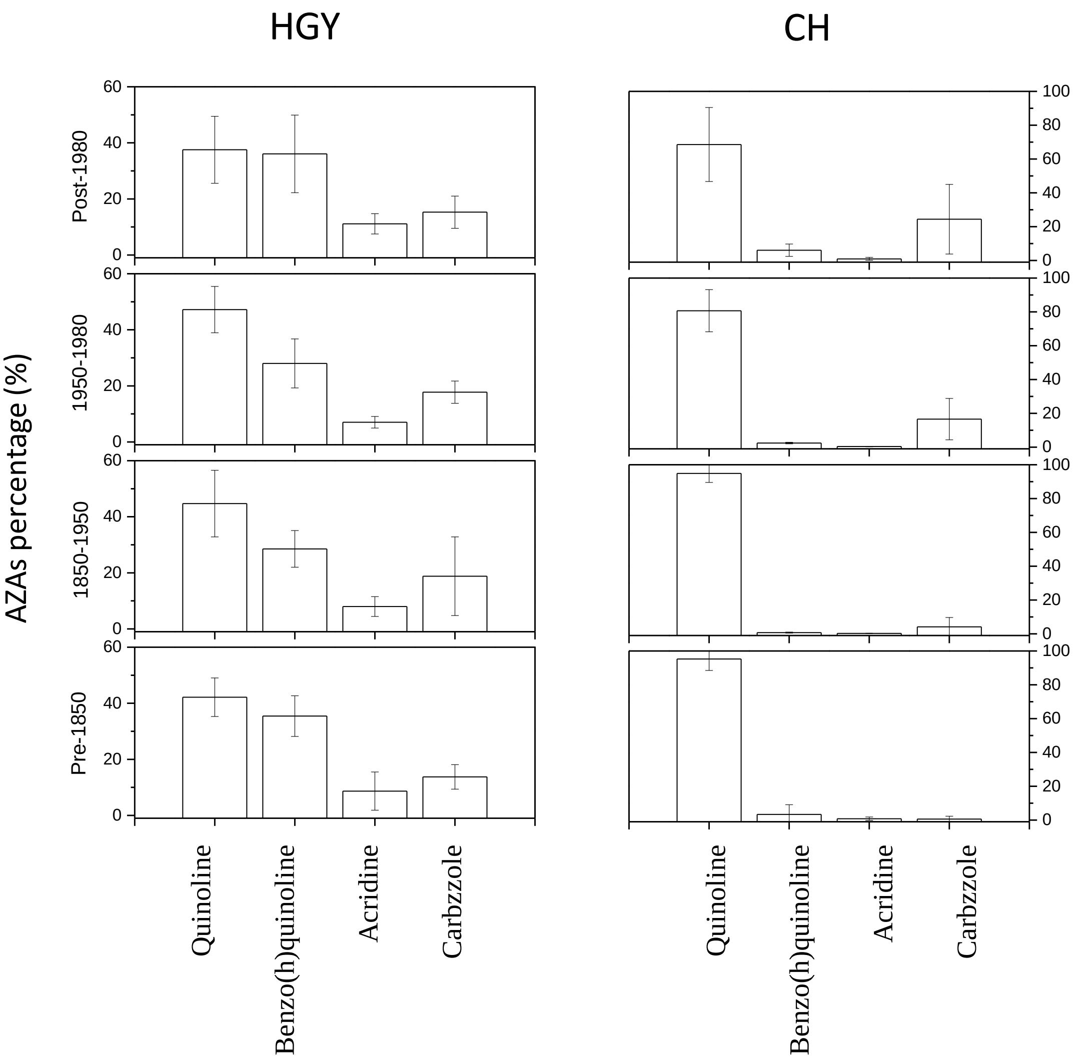

Fig. S4 Azaarenes (AZAs) distribution patterns in different periods for Huangyan Maar Lake (HGY) and Chaohu Lake (CH), respectively.

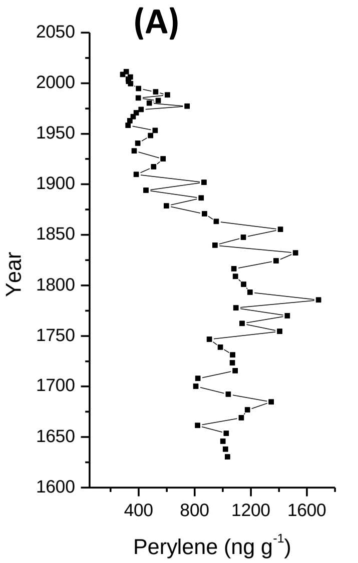

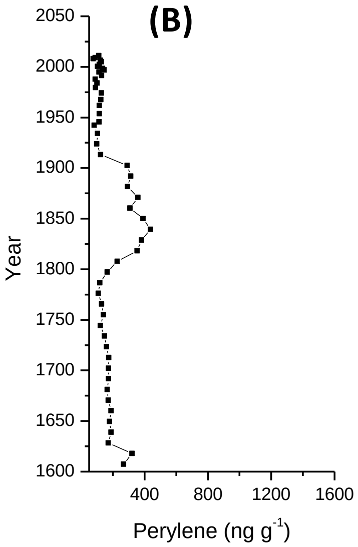

Fig. S5 Concentration profiles of perylene for (A) the Hugang Maar Lake and (B) the Chaohu Lake.

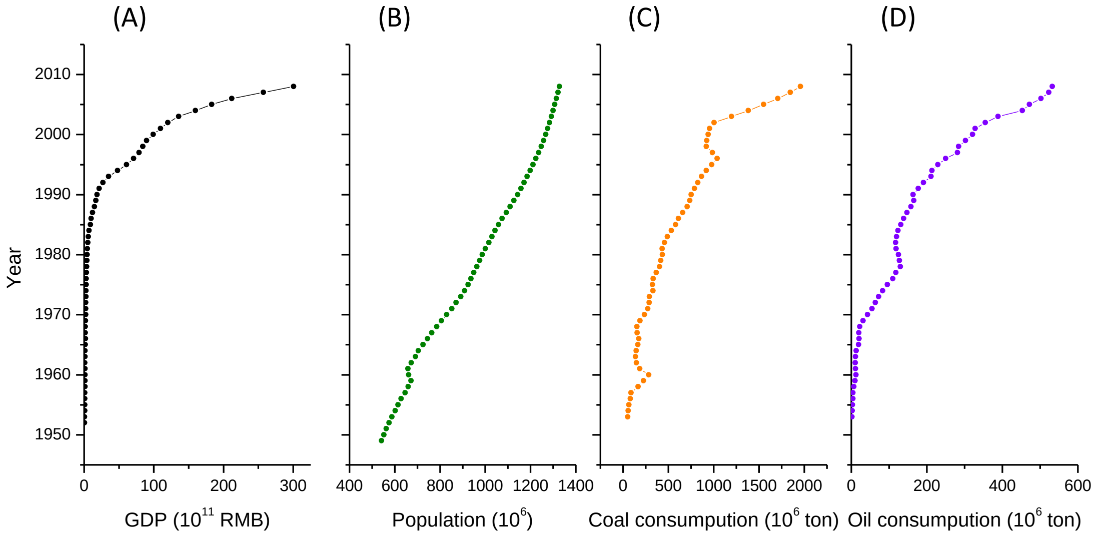

Fig. S6 The increase of economy, populations, and energy consumption. (A) Gross domestic product; (B) Populations; (C) Coal consumption, which has been calculated as standard coal consumption; (D) Oil consumption, which has also been calculated as standard coal consumption.

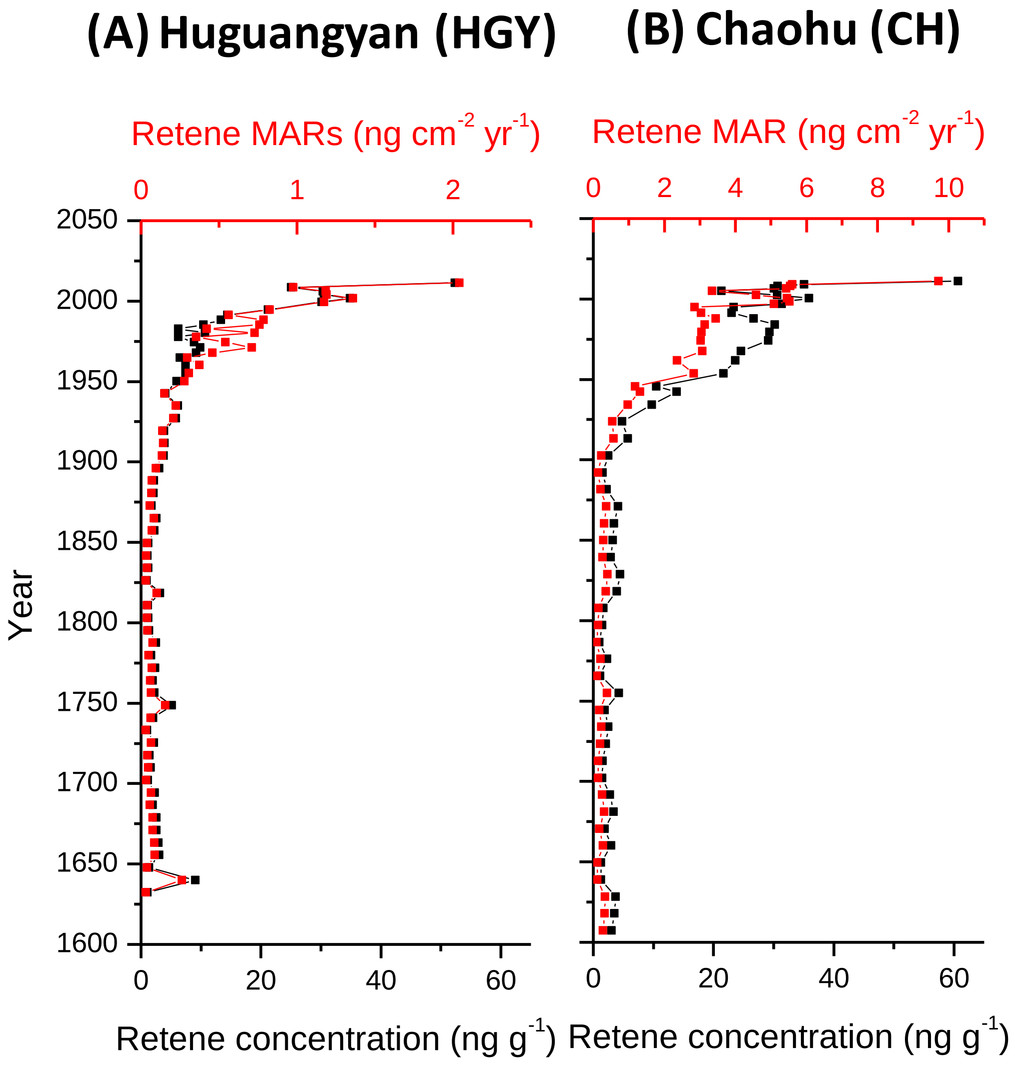

Fig. S7 Similar variations of concentrations and MARs of retene, an indicator of biomass burning, for (A) the Huguangyan Maar Lake and (B) Chaohu Lake

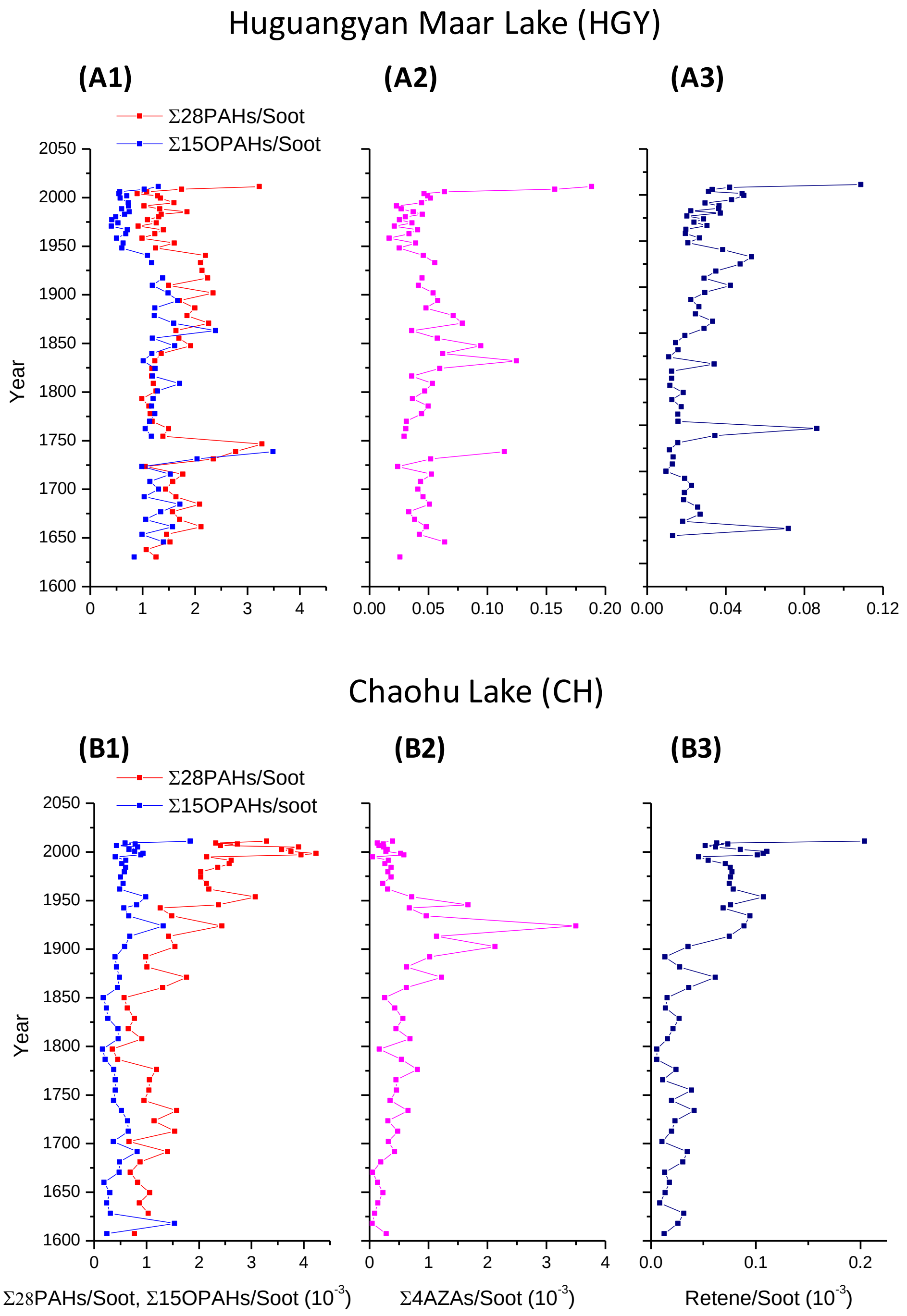

Fig. S8 Historical profiles of ratios of PAHs, oxygenated-PAHs (OPAHs), azaarenes (AZAs, an indicator of human activity), and retene (an indicator of biomass burning) to soot in (A) the Huguangyan Maar Lake and (B) Chaohu Lake. (A1 and B1) ratios of parent-PAHs and oxygenated-PAHs (OPAHs) to soot; (A2 and B2) ratio of azaarenes to soot; (A3 and B3) ratio of retene to soot.

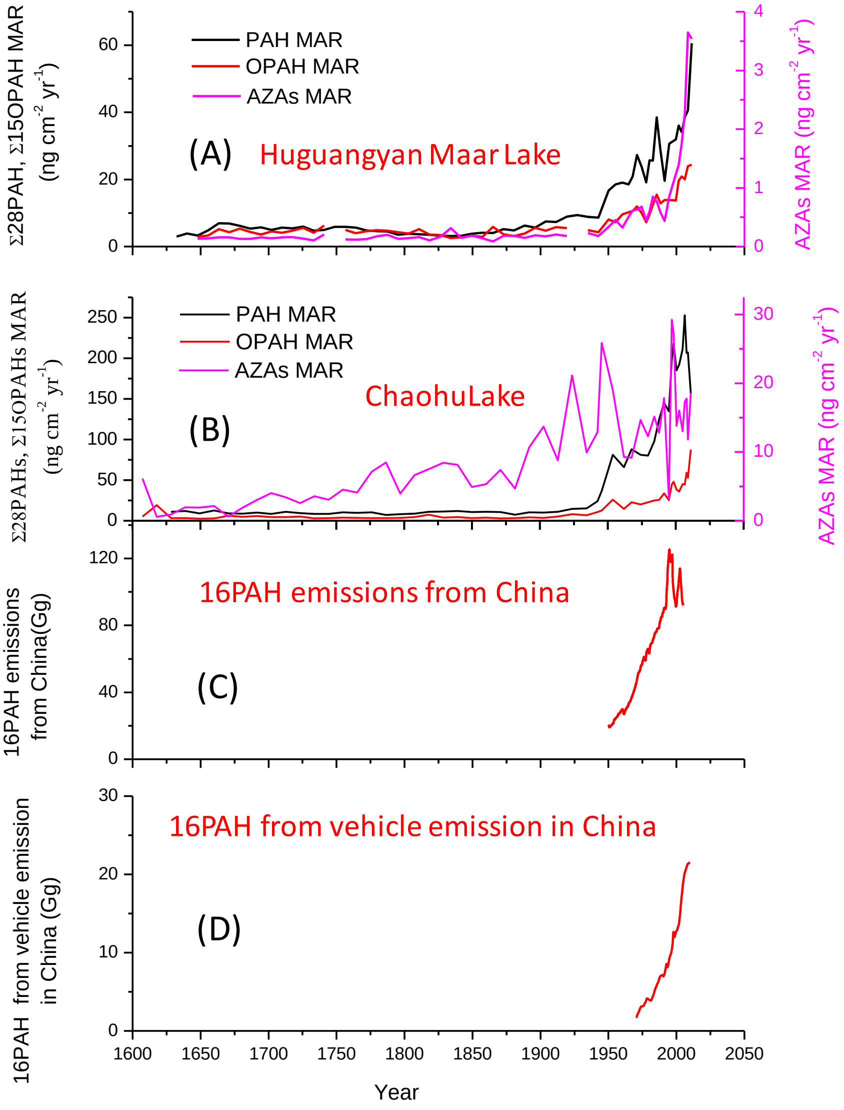

Fig. S9. Comparison of mass accumulation rate (MAR) records of PAH, OPAH, and AZAs from (A) the Huangyan (HGY) Maar Lake and (B) the Chaohu (CH) Lake with the emission inventory records of PAHs from (C) China emissions [13] and from (D) vehicle emissions in China [14]. The abrupt peak for soot MARs in the CH occurring during ~1930s-1940s seems an exception, which is caused by the increase in MARs at that time.

Table S1 Statistical description concentrations of PACs, sum of concentrations of PACs and other sediment properties.

			Huguangyan Maar Lake (HGY)			Chaohu Lake (CH)		
	Abbreviation	Unit	Min.	Max.	Mean	Min.	Max.	Mean
**Azaarenes**	AZAs							
Quinoline	QUI	ng g^{-1}	1.1	57.4	5.5	4.2	224.1	71.3
Benzo(h)quinoline	BhQ	ng g^{-1}	0.5	60.3	6.3	0.1	10.8	2.2
Acridine	ACR	ng g^{-1}	0.05	11.7	1.5	0.1	1.8	0.4
Carbazole	CAR	ng g^{-1}	0.1	83.0	3.1	n.d.[2]	80.6	16.2
**Oxygenated PAHs**	**OPAHs**							
1-Indanone	1-IND	ng g^{-1}	2.8	127.9	15.7	n.d.	42.5	13.2
1,4-Naphthoquinone	1,4-NQ	ng g^{-1}	4.0	568.6	41.7	n.d.	4.5	0.7
1-Naphthaldehyde	1-NALD	ng g^{-1}	0.3	15.9	2.8	n.d.	4.3	0.8
2-Biphenylcarboxaldehyde	2-BPCA	ng g^{-1}	0.9	238.0	9.3	n.d.	8.3	2.9
9-Fluorenone	9-FLUO	ng g^{-1}	4.8	526.0	29.1	n.d.	42.3	10.4
1,2-Acenaphthenequinone	1,2-ACEQ	ng g^{-1}	3.2	421.5	43.5	n.d.	53.7	8.5
9,10-Anthraquinone	9,10-AQ	ng g^{-1}	0.7	31.9	10.3	n.d.	96.1	13.4
1,8-Naphtalic anhydride	1,8-NA	ng g^{-1}	11.1	587.5	43.9	n.d.	22.2	1.0
4H-Cyclopenta(def)phenanthrenone	4-CPHE	ng g^{-1}	0.1	167.9	7.0	n.d.	29.6	1.5
2-Meth-9,10-anthraquinone	2-MAQ	ng g^{-1}	0.2	40.4	6.4	n.d.	1.0	0.1
Benzo(a)florenone	BaFLU	ng g^{-1}	0.9	14.9	3.8	n.d.	18.5	4.0
7H-Benzo(de)anthracen-7-one	BdeAQ	ng g^{-1}	6.5	79.9	23.0	n.d.	61.3	13.5
Benzo(a)anthracene-7,12-dione	BaAQ	ng g^{-1}	0.7	28.1	6.7	n.d.	196.3	12.7
5,12-Naphthacenequinone	5,12-NQ	ng g^{-1}	0.1	33.4	3.9	n.d.	27.0	5.1
6H-Benzo(c,d)pyren-6-one	BcdPQ	ng g^{-1}	1.8	45.3	8.1	n.d.	106.0	23.6
**Polycyclic aromatic hydrocarbons**	**PAHs**							
1,2,3,4-Tetrahydronapthalene	TH-NAPH	ng g^{-1}	0.02	4.6	0.7	n.d.	1.4	0.3
Naphthalene	NAPH	ng g^{-1}	3.3	69.6	16.7	4.7	48.1	17.8
2-Methylnaphthalene	2-MNAPH	ng g^{-1}	0.6	53.7	4.1	1.8	29.0	7.8
1-Methylnaphthalene	1-MNAPH	ng g^{-1}	0.5	96.6	6.6	1.5	30.8	7.5
Biphenyl	BIPH	ng g^{-1}	4.1	99.0	11.8	1.6	32.1	10.0
1,3-Dimethylnaphthalene	1,3-DMNAPH	ng g^{-1}	2.6	275.4	17.6	7.6	183.3	45.9
Acenaphthylene	ACEY	ng g^{-1}	0.4	9.6	1.9	0.2	10.0	2.6
Acenaphthene	ACEN	ng g^{-1}	0.2	6.1	1.3	0.3	30.8	6.3
Fluorene	FLUO	ng g^{-1}	1.5	42.2	8.5	1.4	43.1	10.4
Phenanthrene	PHE	ng g^{-1}	6.9	194.2	51.0	5.4	142.5	42.8
Anthracene	ANT	ng g^{-1}	2.2	41.1	10.5	1.1	42.7	8.8
2-Methylphenanthrene	2-MPHE	ng g^{-1}	2.5	70.7	11.7	1.8	67.2	14.0
3,6-Dimethylphenanthrene	3,6-DMPHE	ng g^{-1}	0.2	10.6	1.9	0.9	67.2	16.1
Fluoranthene	FLUA	ng g^{-1}	4.0	57.8	18.3	1.4	47.6	14.6

Pyrene	PYR	ng g^{-1}	5.0	72.7	20.4	1.8	91.2	27.7
Retene	RET	ng g^{-1}	0.9	52.4	7.3	1.0	60.7	12.7
Benzo(a)anthracene	BaA	ng g^{-1}	1.4	19.8	4.7	1.6	100.1	17.0
Chrysene+Triphenylene	CHR	ng g^{-1}	1.8	120.0	8.2	0.8	57.6	11.2
Benzo(bjk)fluoranthene	BbjkF	ng g^{-1}	11.8	102.3	33.6	5.5	741.7	65.6
Benzo(e)pyrene	BeP	ng g^{-1}	3.1	36.2	14.6	1.9	194.4	33.1
Benzo(a)pyrene	BaP	ng g^{-1}	1.0	26.9	7.4	0.5	290.9	33.9
Perylene	PER	ng g^{-1}	287.8	1683.3	808.7	75.5	438.1	176.2
Indeno (1,2,3-cd)pyrene	IcdP	ng g^{-1}	4.4	49.9	17.1	2.0	313.7	25.5
Dibenzo(a,h)anthracene	DahA	ng g^{-1}	1.1	19.5	4.5	0.7	62.6	12.2
Benzo(ghi)perylene	BghiP	ng g^{-1}	3.2	69.0	22.4	0.9	274.5	30.8
Coronene	COR	ng g^{-1}	0.1	0.8	0.3	8.6	90.5	32.0
Sum of azaarenes	∑4AZAs	ng g^{-1}	2.1	93.5	12.0	6.3	230.9	82.6
sum of oxygenated-PAHs	∑15OPAHs	ng g^{-1}	68.8	626.5	194.2	28.2	546.9	125.5
Sum of polycyclic aromatic hydrocarbons	∑29PAHs	ng g^{-1}	586.5	1867.5	1124.9	195.7	1520.2	617.2
∑PAHs without perylene	∑28PAHs	ng g^{-1}	96.5	1554.1	316.1	78.2	1397.8	449.7
Low molecular weight parent PAHs	LMW-PAHs	ng g^{-1}	19.6	362.9	89.9	15.6	273.5	89.3
High molecular weight parent PAHs without perylene	HMW-PAHs	ng g^{-1}	46.9	528.4	164.6	28.2	754.6	242.8
Combustion-derived PAHs	∑COMB-PAHs[1]	ng g^{-1}	44.6	452.5	146.7	26.9	717.2	224.3
LMW-/HMW-PAHs			0.2	1.0	0.6	0.3	1.5	0.6
∑15OPAHs/∑28PAHs			0.4	1.5	0.7	0.2	0.7	0.4
∑COM-PAHs/∑28PAHs			0.3	0.6	0.5	0.2	0.6	0.4
Total nitrogen	TN	mg g^{-1}	1.8	7.1	3.1	0.5	2.8	1.1
Inorganic carbon	IC	mg g^{-1}	0.1	0.5	0.2	0.2	0.5	0.3
Total organic carbon	TOC	mg g^{-1}	13.8	62.4	26.3	2.8	15.7	6.3
Total sulfur	TS	mg g^{-1}	0.5	7.1	1.6	0.1	1.0	0.3
TOC/TN	C/N	-	7.3	9.1	8.3	4.6	8.3	5.9
Black carbon	BC	mg g^{-1}	0.3	1.7	0.6	0.2	1.0	0.5
Char		mg g^{-1}	0.1	0.8	0.4	0.1	0.5	0.2
Soot		mg g^{-1}	0.1	1.0	0.2	0.1	0.6	0.2

1. ∑COMB-PAHs: Combustion derived PAHs = sum of FLUA, PYR, BaA, CHR, BbjkF, BeP, BaP, IcdP and BghiP;

2. n.d. not detected.

Table S2. Pearson correlation coefficients between carbon fractions and PACs in both Huguangyan Maar Lake and Chaohu Lake.

	∑28PAHs	∑15OPAH	∑AZAs	Retene
	Huguang Maar Lake (HGY)			
ECT	0.884**	0.908**	0.783**	0.869**
Char	0.824**	0.872**	0.774**	0.803**
Soot	0.857**	0.860**	0.724**	0.850**
	Chaohu Lake (CH)			
ECT	0.897**	0.814**	0.233	0.814**
Char	0.738**	0.733**	0.155	0.688**
Soot	0.884**	0.756**	0.254	0.788**

**. Correlation is significant at the 0.01 level (2-tailed).
